# Uridine diphosphate drives myeloid differentiation and functional reprogramming through dynamic transcriptional network

**DOI:** 10.3389/fimmu.2026.1743389

**Published:** 2026-01-30

**Authors:** Caterina Giordano, Debora Gentile, Emilio Straface, Raffaella Gallo, Costanza Maria Cristiani, Antonio Abatino, Arianna Pastore, Marilena Celano, Alessandro Arcucci, Francesco Albano, Geppino Falco, Claudia Veneziano, Gianluca Santamaria, Ilenia Aversa, Lisa Isdraele Romano, Camillo Palmieri, Giuseppe Fiume

**Affiliations:** 1 Department of Experimental and Clinical Medicine, University of Catanzaro “Magna Graecia”, Catanzaro, Italy; 2 Neuroscience Research Center, Department of Medical and Surgical Sciences, University Magna Graecia of Catanzaro, Catanzaro, Italy; 3 Department of Pharmacy, University of Naples Federico II, Naples, Italy; 4 Department of Life Sciences, University of Catanzaro “Magna Graecia”, Catanzaro, Italy; 5 Department of Public Health, University of Naples “Federico II”, Naples, Italy; 6 Department of Biology, University of Naples “Federico II”, Naples, Italy

**Keywords:** dendritic cells, innate immunity, monocyte differentiation, purinergic signaling, uridine diphosphate (UDP)

## Abstract

**Background:**

Extracellular nucleotides regulate immune responses through purinergic signaling. Uridine diphosphate (UDP), a pyrimidine-derived metabolite, has been shown to accumulate in the tumor microenvironment and modulate T cell activation. However, its effects on human myeloid cells remain poorly understood. Since monocytes represent key precursors for macrophages and dendritic cells, we investigated whether UDP could influence their proliferative and differentiation potential within peripheral blood mononuclear cells (PBMCs).

**Methods:**

Freshly isolated PBMCs were stimulated with UDP, and CD14^+^ cell proliferation was analyzed using CFSE staining and flow cytometry. The impact of UDP on dendritic differentiation was evaluated in PBMC cultures and in purified CD14^+^ monocytes exposed to IL-4 and GM-CSF, in the presence or absence of UDP. Phagocytic and efferocytic activities were assessed using fluorescently labeled E. coli and apoptotic HeLa cells, respectively. Transcriptomic profiling of PBMCs stimulated with UDP for 2, 6, or 24 hours was performed using the NanoString Human Immunology Panel.

**Results:**

UDP markedly suppressed CD14^+^ monocyte proliferation and promoted the generation of HLA-DR^+^CD11c^+^ dendritic-like cells. In purified monocytes, UDP enhanced IL-4/GM-CSF-driven differentiation into CD14^-^CD16^-^HLA-DR^+^CD11c^+^ monocyte-derived dendritic cells (moDCs). Functionally, UDP increased both bacterial phagocytosis and efferocytosis. Transcriptomic analysis revealed eight gene clusters with distinct temporal expression patterns, driven by transcription factors such as NF-κB, RUNX3, BATF, and IRF5, indicating coordinated modulation of inflammatory, antigen-presentation, and regulatory pathways.

**Conclusion:**

Our findings identify UDP as a potent immunomodulatory metabolite that restricts monocyte proliferation while promoting differentiation into dendritic-like cells with enhanced phagocytic capacity. UDP engages complex transcriptional programs that integrate innate activation with adaptive immune regulation, highlighting its potential role in immune homeostasis and inflammation control.

## Introduction

Extracellular nucleotides have emerged as key immunometabolic signals that influence the function, differentiation, and plasticity of immune cells (1–5). Among these, uridine diphosphate (UDP) has gained increasing attention as a potent extracellular signaling molecule, capable of modulating immune responses through the activation of the G protein-coupled receptor P2Y6 (P2RY6) (6–9). This receptor is prominently expressed on myeloid cells, including monocytes, macrophages, dendritic cells (DCs), and microglia, and mediates a variety of immune-regulatory functions upon ligand binding (10–12). Our previous work demonstrated that UDP accumulates within the tumor interstitial fluid (TIF) of melanoma-bearing mice and acts as a potent immune modulator capable of enhancing T-cell activation, driving Th1/Th17 polarization, and promoting anti-tumor immunity (4). Specifically, we showed that UDP treatment increased the frequency of CD8^+^ and CD4^+^ tumor-infiltrating lymphocytes (TILs) and activated NF-κB and STAT signaling pathways in CD4^+^ T-cells (4). Moreover, intra-tumoral administration of UDP reduced tumor growth and necrotic areas, highlighting its potential as an immunometabolic mediator capable of shaping anti-tumor immune responses (4). These findings raised the possibility that extracellular UDP might exert broader immunoregulatory effects on innate and adaptive immune cells beyond the tumor microenvironment. However, subsequent studies have revealed a more complex and context-dependent role for UDP. Scolaro et al., uncovered a paradoxical function of UDP in promoting tumor immune evasion. They showed that UDP production in cancer cells, through the activity of cytidine deaminase (CDA), sustained immunosuppressive programming of tumor-associated macrophages (TAMs) via P2RY6 signaling. This pathway limited cytotoxic T cell infiltration and conferred resistance to immune checkpoint inhibitors such as anti-PD-1 (13–15). Genetic or pharmacologic inhibition of either CDA in tumor cells or P2RY6 in TAMs reversed the immunosuppressive TME and restored sensitivity to immunotherapy. Consistent with this, additional studies have shown that UDP–P2RY6 signaling plays multifaceted roles in immune modulation and disease. In cancer, aberrant expression of the pyrimidinergic receptor P2RY6 in tumor cells has been identified as a key mechanism of immune evasion (16). Tumor-intrinsic P2RY6 enhances prostaglandin E_2_ synthesis through Gq/phospholipase C-β signaling, fostering an immunosuppressive tumor microenvironment and resistance to PD-1 blockade, whereas genetic ablation of P2RY6 reprograms the tumor milieu toward an inflamed, immune-active state (16). Importantly, global deletion of P2RY6 in mice does not compromise viability, underscoring its potential as a selective target for precision immunotherapy (16). These findings underscored the dual role of UDP–P2RY6 signaling, acting either as an immune activator or suppressor depending on the cellular and molecular context (4, 13, 16). The immune-regulatory functions of UDP and P2RY6 extend beyond cancer and have also been implicated in the central nervous system (CNS) (17). Using a genetically encoded UDP sensor (GRABUDP1.0), it has been showed that seizures and excitotoxicity trigger rapid extracellular UDP release in the brain (18). This event activated microglial P2RY6 and induced calcium-dependent signaling associated with lysosome biogenesis, enhanced phagocytosis of neurons, and increased NF-κB-driven cytokine production. Notably, genetic ablation of P2RY6 reduced microglial phagocytic engagement and neuroinflammatory damage, resulting in improved cognitive function in epileptic mice. These data provide compelling evidence that the UDP–P2RY6 axis critically regulates neuroimmune interactions during pathological states. Adding further complexity, a study by Timmerman et al., demonstrated that P2RY6 signaling amplifies Toll-like receptor (TLR)-mediated pro-inflammatory responses, particularly in microglia (19). Their transcriptomic and functional analyses showed that P2RY6 enhances TLR1/2/4/5/8-driven cytokine production via NF-κB, IRF, and NFAT transcription factors. Interestingly, P2RY6 antagonism led to a robust increase in the expression of heat shock proteins, suggesting a protective response under inflammatory or homeostatic conditions. These results emphasize that P2RY6 acts as a signal integrator in innate immunity, modulating both immune activation and stress responses (19). Collectively, these studies highlight the multifaceted role of the UDP–P2RY6 axis in immunity, operating as a double-edged sword that can either promote immune activation or immunosuppression depending on tissue-specific and environmental cues. Despite this growing body of evidence, the role of extracellular UDP in human myeloid cell fate decisions, particularly during monocyte-to-DC differentiation and functional programming, remains poorly defined. In the present study, we investigated the effects of UDP on human PBMCs using an integrated experimental and transcriptomic approach. We demonstrate that UDP suppresses the proliferation of CD14^+^ monocytes and promotes their differentiation into HLA-DR^+^CD11c^+^ dendritic-like cells. Functionally, UDP enhances both bacterial phagocytosis and efferocytosis, indicating a global reprogramming of myeloid cells toward a differentiated and functionally competent state. Finally, transcriptomic profiling revealed dynamic, temporally distinct UDP-induced gene expression programs regulated by transcription factors such as NF-κB, BATF, and IRF5. Together, these findings identify UDP as a multifaceted immunomodulatory metabolite capable of orchestrating cellular differentiation, innate activation, and transcriptional rewiring in human PBMCs.

## Materials and methods

### Source of human mononuclear cells

Peripheral blood mononuclear cells (PBMC) were freshly isolated from leukocyte concentrate (buffy-coat) collected from healthy donor volunteers at Azienda Ospedaliera Universitaria “R. Dulbecco” in Catanzaro (Calabria, Italy). Ethical clearance for the use of human subjects was obtained from the designated health care facility. Written informed consent was obtained from each person after receiving information about the use of their blood samples. Biological material was anonymized.

### Isolation of human peripheral blood mononuclear cells and purification of monocytes

PBMCs were separated from the buffy coat within 24 hours of obtaining blood samples using a density gradient (Ficoll-Histopaque-1077, Sigma-Aldrich, Darmstadt, Germany), following the manufacturer’s protocol. Next, in order to optimize the monocyte isolation method, we isolated the target cells using a kit involving CD14+ cell-specific magnetic beads (CD14+ MicroBeads human, Miltenyi Biotec, Bergisch Gladbach, Germany), following the manufacturer’s protocol.

### MoDCs differentiation

For differentiation, we relied on a previously described protocol (20). Briefly, after purification, cell density was adjusted to 0.5 × 10^6^ cells/mL. CD14+ cells were cultured in 24-well tissue culture plates, resuspended in RPMI-1640 culture medium (Sial, Rome, Italy) supplemented with 10% fetal bovine serum (FBS) (Termofisher, Waltham, Massachusetts, USA), 1% Penicillin-Streptomicin, and Glutamine (Termofisher, Waltham, Massachusetts, USA). MoDC differentiation medium was obtained by supplementing each culture medium described above with 400 IU/mL recombinant human GM-CSF, 100 IU/mL rhIL-4 (PeproTech Inc., Waltham, Massachusetts, USA) and 100 µM UDP (Sigma, Darmstadt, Germania). After 48 hours, half the total volume of each well was removed and new cytokine culture medium was added for an additional 2 days. Undifferentiated monocytes (negative control) were arranged in parallel wells and received their respective cytokine-free culture medium. Furthermore, the wells were prepared without the addition of UDP.

### Quantitative real-time PCR

Total RNA was extracted from cells by using the TRIzol reagent (Invitrogen, California, USA); RNA aliquots (500 ng) were reverse transcribed using High- Capacity cDNA Reverse Transcription Kit (Thermofisher, Waltham, Massachusetts, USA), according to the manufacturer’s protocol. Real-time PCR was performed with the YourSial Green Mix (Sial, Rome, Italy) and carried out with the Quant Studio 3 (Thermofisher, Waltham, Massachusetts, USA) under the following conditions: 95°C, 1 min; (94°C, 10 s; 60°C, 30 s) × 40. Primers used in reaction was: Actin (Fw: ACAGAGCCTCGCCTTTGC; Rv: CCACCATCACGCCCTGG), CD80 (Fw: CAACCACAGCTTCATGTGTCTC; Rv: CATCTTGGGGCAAAGCAGTAG) and CD40 (Fw: ACCCTTGGACAAGCTGTGAG; Rv: TGGCTTCTTGGCCACCTTTT). Reactions were carried out in triplicate, and gene expression levels were calculated relative to Actin mRNA levels as endogenous control. Relative expression was calculated as 2 (Ct gene under investigation−Ct Actin).

### Flow cytometric analysis

To verify target cell isolation yields, we analyzed target cell frequency based on morphological characteristics (FSC vs. SSC) and expression of a specific monocyte marker (CD14) by flow cytometry, as described in (21, 22). For flow cytometry procedures, specific commercial antibodies were diluted in FACS buffer (PBS in 1% BSA and 0.05% NaN3). The surface markers used were anti-human CD14-BV766, CD14-PE, CD16-BV421, HLA-DR-APC, HLA-DR-PerCP, CD11c-PE, CD11c-BV605, and CD11b-PE-Cy-7 (BD Biosciences, New Jersey, USA). Isolated monocytes and undifferentiated monocytes were incubated for 30 minutes at 4 °C with viability stain (Fixable Viability Stain 780, BD Biosciences, New Jersey, USA). The cells were then washed twice in ice-cold FACS buffer and subsequently incubated for 15 minutes at 4 °C with the antibodies listed above. Finally, the cells were washed twice in ice-cold FACS buffer and resuspended in 300 μL of FACS buffer. For flow cytometry analyses, at least 50,000 events were acquired for each sample. All experiments were independently repeated a minimum of three times. The exact number of biological replicates (n) for each experiment is reported in the corresponding figure legends. Acquisitions were performed with FACS Fortessa X-20 (BD Biosciences, New Jersey, USA) to evaluate monocytes differentiated into immature dendritic cells. Flow cytometry data was analyzed with FlowJo software V10.10.

### Bacterial phagocytosis assay

Phagocytosis assays were performed using DH5α *E. coli* stably transformed with the pCMV6-AC-RFP Mammalian Expression Vector (Origene, Rockville, Maryland, USA) to express red fluorescent protein (RFP). Bacteria were cultured in LB broth at 37°C until mid-logarithmic phase (OD_600_ = 0.6). Aliquots of the bacterial suspension (50 µL, OD_600_ = 0.6) were added to 5 × 10^6^ PBMCs, which had been pretreated or not with 100 µM UDP for 2 h at 37°C in complete RPMI medium. Co-cultures were immediately transferred to a Leica Thunder Imager microscope equipped with a live-cell incubation chamber, maintained at 37°C and 5% CO_2_. Time-lapse imaging was performed at 5, 30, 60, and 90 min after bacterial addition to monitor dynamic phagocytic activity. Phagocytosis was quantified by calculating the Phagocytic Index, defined as the ratio of intracellular red fluorescence intensity to the total number of PBMCs in the field of view. Intracellular RFP signal was quantified using ImageJ software, analyzing at least five random fields per condition across three independent experiments. Representative 90 minutes time-lapse videos of PBMC-bacteria co-cultures in the presence or absence of UDP are provided as **Supplementary Videos S1** (Not treated) and 2 (UDP-treated).

### Efferocytosis assay

Apoptotic HeLa cells were generated by culturing in HBSS for 3 days to induce nutrient-deprivation–mediated apoptosis. Cells were labeled with CellTrace CFSE (1 µM; Invitrogen, California, USA) for 20 min at 37°C, washed extensively, and resuspended in complete medium. PBMCs (5 × 10^6^), pre-incubated with or without 100 µM UDP for 2 h, were co-cultured with 1 × 10^5^ CFSE-labeled apoptotic HeLa cells for 3 h or 6 h in the presence or absence of UDP. At the end of incubation, PBMCs were fixed with 2% paraformaldehyde for 7 min, permeabilized with 0.1% Triton X-100 in PBS for 5 min, and washed in PBS. Cells were incubated with primary antibody against LAMP-1 (clone H4A3, 1:100 dilution, BD Biosciences, New Jersey, USA) for 1 h at room temperature, followed by washing with PBS containing 0.1% Tween (PBS-T) and incubation with Alexa Fluor^®^ 488-conjugated secondary antibody (1:1000 dilution, Invitrogen, Invitrogen, California, USA) for 1 h at room temperature. Nuclei were counterstained with DAPI (1:25000). Immunofluorescence images were acquired with a Leica Stellaris Confocal microscope. Efferocytosis was quantified as the percentage of PBMCs containing CFSE-positive dots colocalizing with LAMP-1 signal, calculated over at least 100 cells per condition using ImageJ software.

### Transcriptomic profiling

Freshly isolated PBMCs (1 × 10^6^ per condition) were cultured in complete RPMI medium and stimulated with 100 µM UDP for 2 h, 6 h, or 24 h. Unstimulated PBMCs were used as controls. All conditions were performed in biological triplicates. At each time point, cells were harvested, and total RNA was extracted using TRIzol reagent (Invitrogen, California, USA) according to the manufacturer’s instructions. RNA concentration and purity were assessed by spectrophotometry (NanoDrop ND-1000, Thermo Scientific, Waltham, Massachusetts, USA). For each sample, 1 µg of total RNA was hybridized with the nCounter^®^ Immunology Panel (Nanostring Technologies, Seattle, Washington, USA), which profiles the expression of about 500 immune-related genes. Hybridization, post-hybridization processing, and digital counting of barcoded probes were performed using the nCounter^®^ Analysis System (Nanostring Technologies, Seattle, Washington, USA) following the manufacturer’s protocol. Data normalization and quality control were carried out using nSolver Analysis Software (Nanostring Technologies, Seattle, Washington, USA). Differential expression analysis was performed using the R Bioconductor package DESeq2 (23). Differentially expressed genes (DEGs) were considered dysregulated with absolute log2(Fold Change) > 1 and padj < 0.05. To investigate patterns of gene expression, hierarchical clustering was performed on the z-score normalized expression matrix using the pheatmap package in R. To extract biologically meaningful gene groups, the resulting gene dendrogram (tree_row) was cut into discrete clusters using the cutree() function. The transcription factor enrichment analysis was performed by ChIP-X Enrichment Analysis 3 (ChEA3) (https://maayanlab.cloud/chea3/) with the integrated rank score. Results from the ChEA3 TF target libraries were ranked based on the p-value from Fisher’s Exact Test, which estimates the significance of overlap between the input gene set and the putative transcriptional targets of each TF.

## Results

### UDP limits proliferation of CD14^+^ monocytes, suggesting a role in enforcing a differentiative state

**Figure 1:**
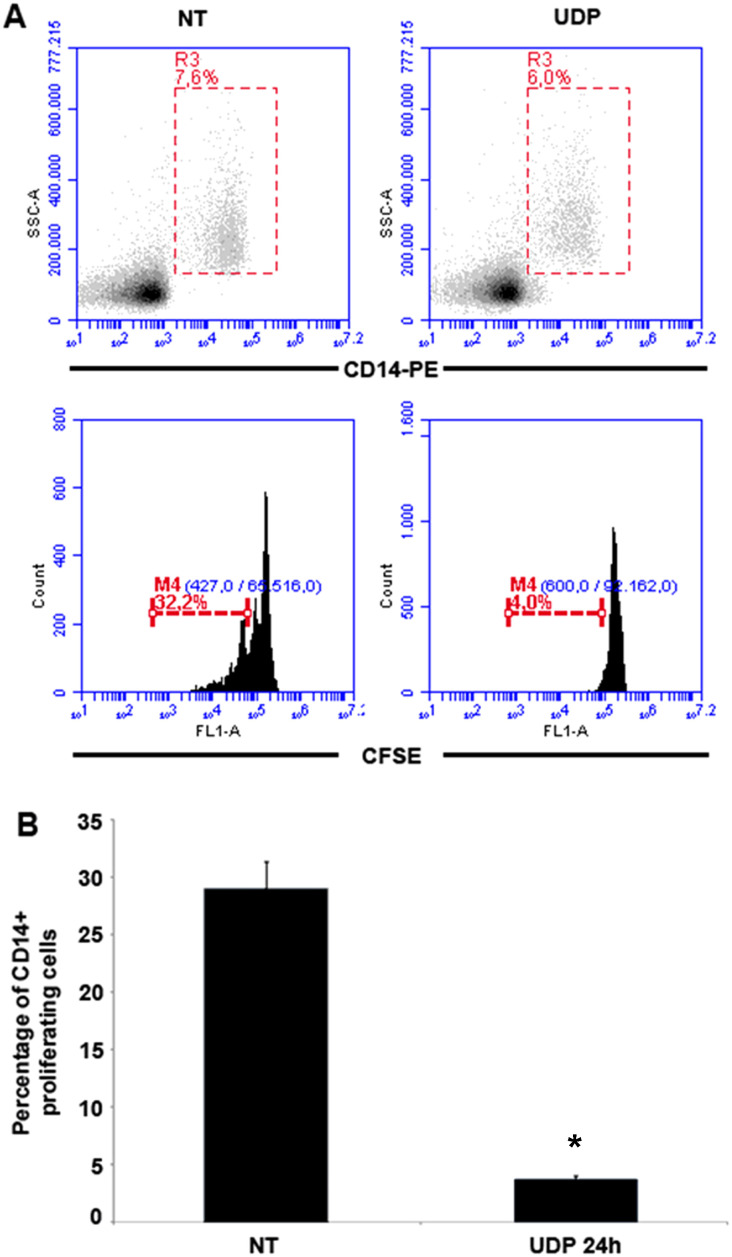
UDP limits the proliferation of CD14+ monocytes. **(A)** Peripheral blood mononuclear cells (PBMCs 3 × 10^6^) were treated with 100 µM UDP and subsequently labeled with CellTrace™ CFSE at a final concentration of 5 µM for 20 minutes at 37°C. Following labeling, cells were cultured *in vitro* for 3 days. Proliferation analysis based on CFSE dilution was performed specifically on the CD14^+^ monocyte population, identified by staining with anti-CD14-PE antibodies and gated accordingly during flow cytometric analysis. **(B)** Percentage of proliferating CD14^+^ monocytes. Data are presented as mean ± SEM (n = 3). Asterisks indicate statistically significant differences compared to the sample treated with UDP for 24 hours, as determined by Student’s t-test (*p < 0.01).

To understand the impact of UDP on human myeloid cell biology, we first investigated whether extracellular UDP influences the proliferative dynamics of peripheral blood mononuclear cells (PBMCs), with particular focus on CD14^+^ monocytes. CD14^+^ cells represent the main monocytic population in PBMCs and serve as precursors for macrophages and dendritic cells under inflammatory or homeostatic conditions. Given the dual role of UDP–P2RY6 signaling in regulating inflammatory responses and shaping immune cell functions in various tissues, including the tumor microenvironment and CNS, we hypothesized that UDP may directly affect monocyte homeostasis, potentially influencing their proliferative capacity and subsequent differentiation fate. To test this hypothesis, freshly isolated PBMCs were labeled with the cell proliferation dye CFSE (carboxyfluorescein succinimidyl ester) and cultured for 72 hours in the presence or absence of 100 µM UDP. CFSE intensity decreases with each cell division, allowing quantification of proliferative activity. After incubation, cells were harvested and stained with anti-CD14 antibodies, and CFSE dilution was assessed by flow cytometry within the CD14^+^ cell population. In untreated control conditions, approximately 32% of the CD14^+^ monocytes exhibited CFSE dilution, indicating active cell proliferation (**Figures 1A, B**). In contrast, in cultures treated with UDP, the percentage of proliferating CD14^+^ cells was dramatically reduced to 4% (**Figures 1A, B**). This suggests that extracellular UDP profoundly suppresses the proliferative activity of CD14^+^ monocytes. Importantly, this effect was observed specifically within the CD14^+^ cell compartment, suggesting that the marked reduction in proliferating cells could result from phenotypic drift, apoptotic loss of monocytes, or a strong inhibition of cell cycle progression induced by UDP. Taken together, these results demonstrate that UDP functions as a negative regulator of monocyte proliferation, possibly engaging purinergic signaling pathways that enforce a quiescent or differentiative state. These findings set the stage for further investigation into the role of UDP in shaping myeloid cell fate and function in the context of immune activation and inflammation.

### UDP promotes the increase of HLA-DR^+^CD11c^+^ dendritic-like cells from PBMCs

**Figure 2:**
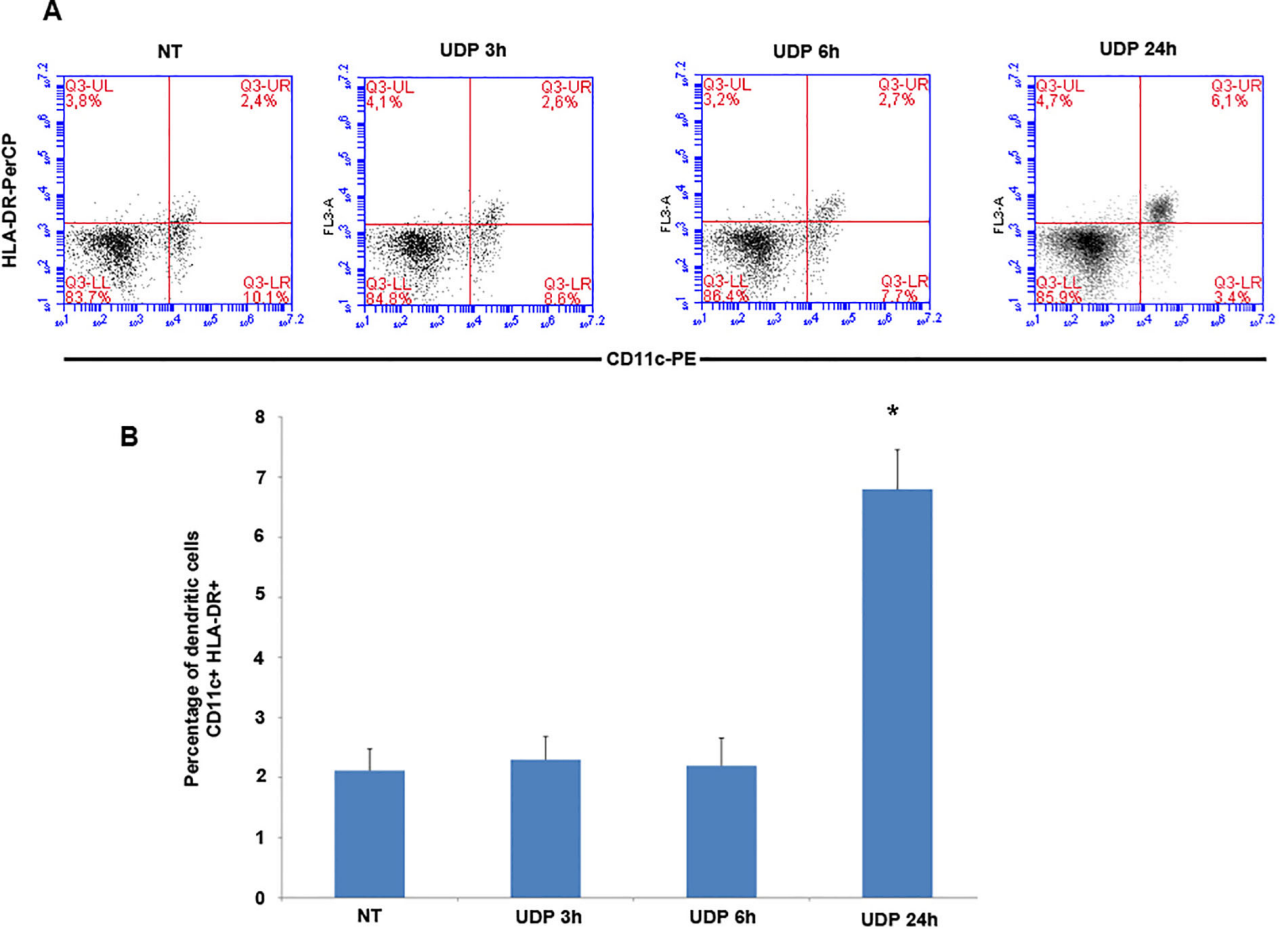
UDP promotes the increase of HLA-DR^+^CD11c^+^ dendritic-like cells in PBMC. **(A)** PBMCs (3 × 10^6^) were treated with 100 µM UDP and subsequently stained with anti-CD11c-PE and anti-HLA-DR-PerCP antibodies. **(B)** Percentage of differentiating CD11c^+^ HLA-DR^+^ cells. Data are presented as mean ± SEM (n = 3). Asterisks indicate statistically significant differences compared to the untreated control, as determined by Student’s t-test (p < 0.01).

Given the strong inhibition of CD14^+^ monocyte proliferation observed upon UDP treatment, we next hypothesized that UDP might modulate the differentiation trajectory of these cells. Monocytes serve as precursors for antigen-presenting cells, including dendritic cells (DCs), particularly under inflammatory or immunomodulatory conditions. In this context, the acquisition of surface markers such as HLA-DR and CD11c is indicative of a transition toward a dendritic cell phenotype. We therefore investigated whether UDP stimulation could promote the generation or accumulation of HLA-DR^+^CD11c^+^ cells from PBMCs *in vitro*. To test this, freshly isolated PBMCs were cultured with or without 100 µM UDP and analyzed by flow cytometry at different time points (3, 6, and 24 hours) to assess the frequency of HLA-DR^+^CD11c^+^ cells. In untreated control conditions, the percentage of HLA-DR^+^CD11c^+^ cells remained around 2%, consistent with the presence of a small steady-state dendritic-like population within PBMCs (**Figures 2A, B**). Interestingly, treatment with UDP for 24 hours significantly increased the frequency of HLA-DR^+^CD11c^+^ cells, reaching approximately 7% of total PBMCs (**Figures 2A, B**). This effect was not observed at earlier time points: UDP stimulation for 3 or 6 hours did not induce any appreciable change in the proportion of HLA-DR^+^CD11c^+^ cells compared to controls. These findings suggest that the effect of UDP on dendritic-like cell generation is time-dependent and likely reflects a differentiation process requiring sustained exposure. In light of the observed decrease in monocyte proliferation, this increase in HLA-DR^+^CD11c^+^ cells may reflect a UDP-driven skewing of monocyte fate toward antigen-presenting phenotypes rather than expansion through proliferation.

### UDP promotes monocyte-to-dendritic cell differentiation in an IL-4/GM-CSF culture system

**Figure 3:**
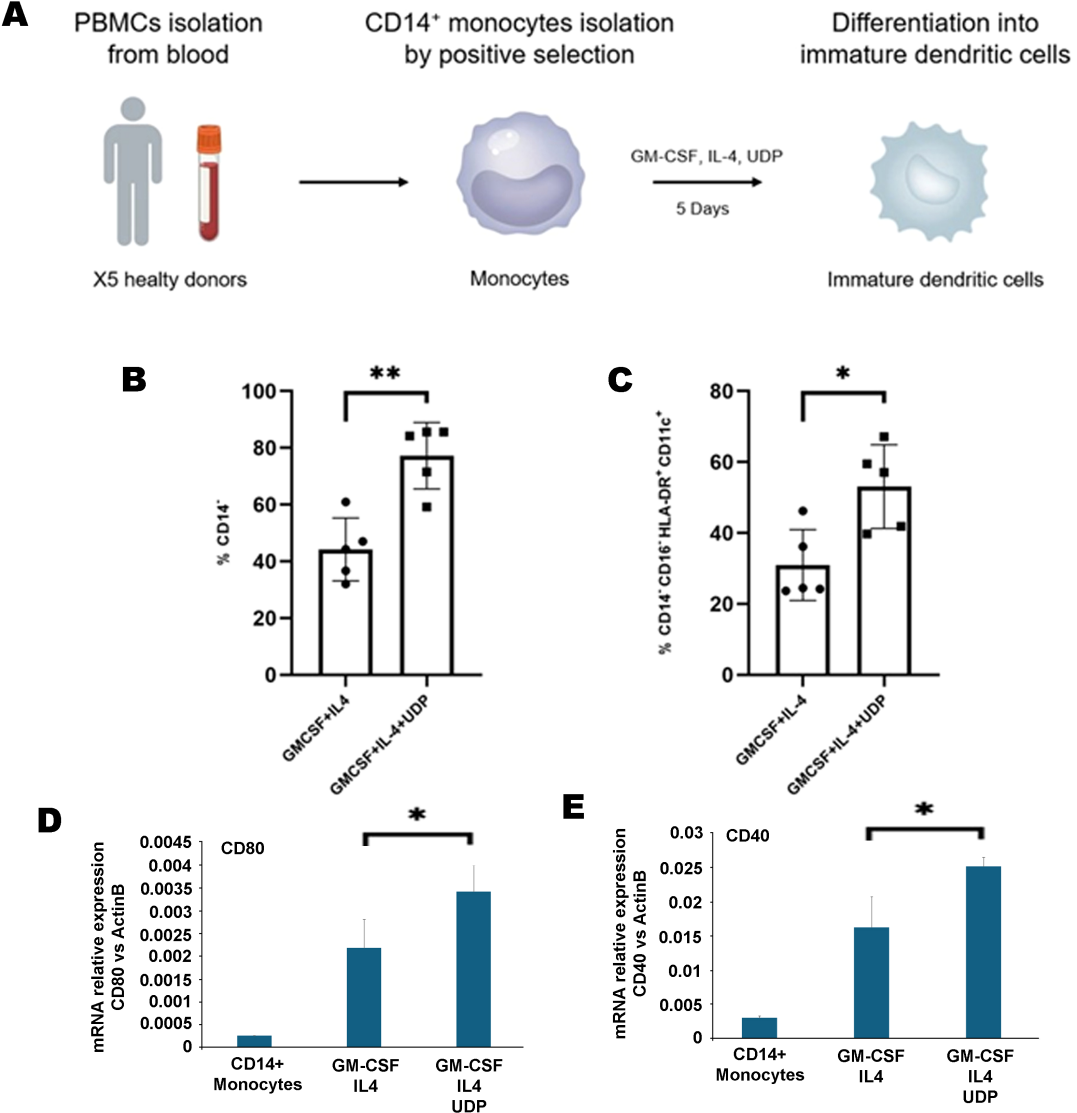
Workflow for the generation of monocyte-derived immature dendritic cells (moDCs) from peripheral blood and modulatory effect of UDP on CD14^+^ cell differentiation and activation phenotype. **(A)** Schematic representation of the experimental workflow: peripheral blood mononuclear cells (PBMCs) were isolated from five healthy donors, starting from approximately 50 × 10^6^ PBMCs for donor. From each preparation, 15 × 10^6^ CD14^+^ monocytes were obtained by positive selection. For differentiation into immature dendritic cells, 5 × 10^5^ CD14^+^ cells were seeded for well in 1 mL of RPMI medium in 24-well plates and cultured for 5 days in the presence of GM-CSF (400 IU/mL) and IL-4 (100 IU/mL), either with or without UDP (100 µM) supplementation. **(B)** Flow cytometry analysis of the percentage of CD14^-^ cells after differentiation in the absence or presence of UDP, showing a significant increase in UDP-treated conditions (**p < 0.01). **(C)** Flow cytometry analysis of CD14^-^CD16^+^HLA-DR^+^CD11c^+^ cells in control versus UDP-treated cultures, highlighting a significant enrichment upon UDP supplementation (*p < 0.05). Data are presented as mean ± SEM of independent experiments performed with cells from five donors. **(D, E)** For the mRNA expression analysis of CD80 and CD40 genes, 2x10^6^ monocytes for each experimental condition were isolated from PBMCs by positive selection using anti-CD14 microbeads. CD14^+^ monocytes were differentiated for 5 days in the presence of GM-CSF (400 U/ml) and IL-4 (100 U/ml), with or without UDP (100 µM). Total RNA was then extracted, reverse-transcribed into cDNA, and analyzed by real-time PCR to assess CD80 **(D)** and CD40 **(E)** expression levels. Data are presented as mean ± SEM (n = 3). Asterisks indicate statistically significant differences compared to the untreated control, as determined by Student’s t-test (*p* < 0.01).

To confirm whether UDP actively promotes the differentiation of monocytes into dendritic cells, we employed a well-established *in vitro* model of monocyte-derived dendritic cell (moDC) generation. CD14^+^ monocytes were isolated from human PBMCs using a negative selection strategy (Miltenyi Biotec) and subsequently cultured for five days in the presence of recombinant human IL-4 and GM-CSF, with or without the addition of 100 µM UDP (**Figure 3A**). This system mimics the cytokine milieu required for DC commitment and allows precise assessment of factors influencing monocyte differentiation. This method allowed us to specifically assess whether UDP could modulate the differentiation process independently of other cell types present in PBMC cultures. Flow cytometry analysis revealed that UDP treatment led to a significant reduction in CD14 expression, indicative of monocyte-to-non-monocyte transition. Specifically, the percentage of CD14^-^ cells increased from approximately 40% in the control condition to 75% in UDP-treated cultures (**Figure 3B**, **Supplementary Figure S1**), suggesting enhanced exit from the monocytic state. Moreover, phenotypic analysis of the CD14^-^ cell compartment showed a substantial increase in cells displaying a dendritic cell-like immunophenotype, defined as CD14^-^ CD16^-^ CD11c^+^ HLA-DR^+^. This population was significantly expanded in the presence of UDP compared to control conditions (**Figure 3C**, **Supplementary Figure S1**), supporting the hypothesis that UDP actively enhances the differentiation of monocytes toward a dendritic lineage in a cytokine-driven context. These findings corroborate our previous observations in total PBMC cultures and indicate that extracellular UDP not only inhibits monocyte proliferation but also promotes their differentiation into immunocompetent dendritic cells under appropriate inflammatory cues. To further determine whether UDP-induced phenotypic changes were accompanied by the acquisition of dendritic cell maturation features, we next evaluated the expression of key co-stimulatory molecules associated with DC functional competence. In particular, the expression of CD80 and CD40 was analyzed by quantitative real-time PCR in monocytes differentiated for five days with GM-CSF and IL-4 in the presence or absence of UDP. Both CD80 and CD40 transcripts were significantly upregulated in UDP-treated cultures compared to cells differentiated with GM-CSF and IL-4 alone (**Figures 3D, E**), indicating that UDP not only promotes the phenotypic transition toward a dendritic cell-like state but also enhances the expression of maturation-associated markers. These data suggest that UDP contributes to the functional reprogramming of monocyte-derived dendritic cells within a cytokine-driven differentiation framework.

### UDP enhances the phagocytic and efferocytic activity of PBMCs

**Figure 4:**
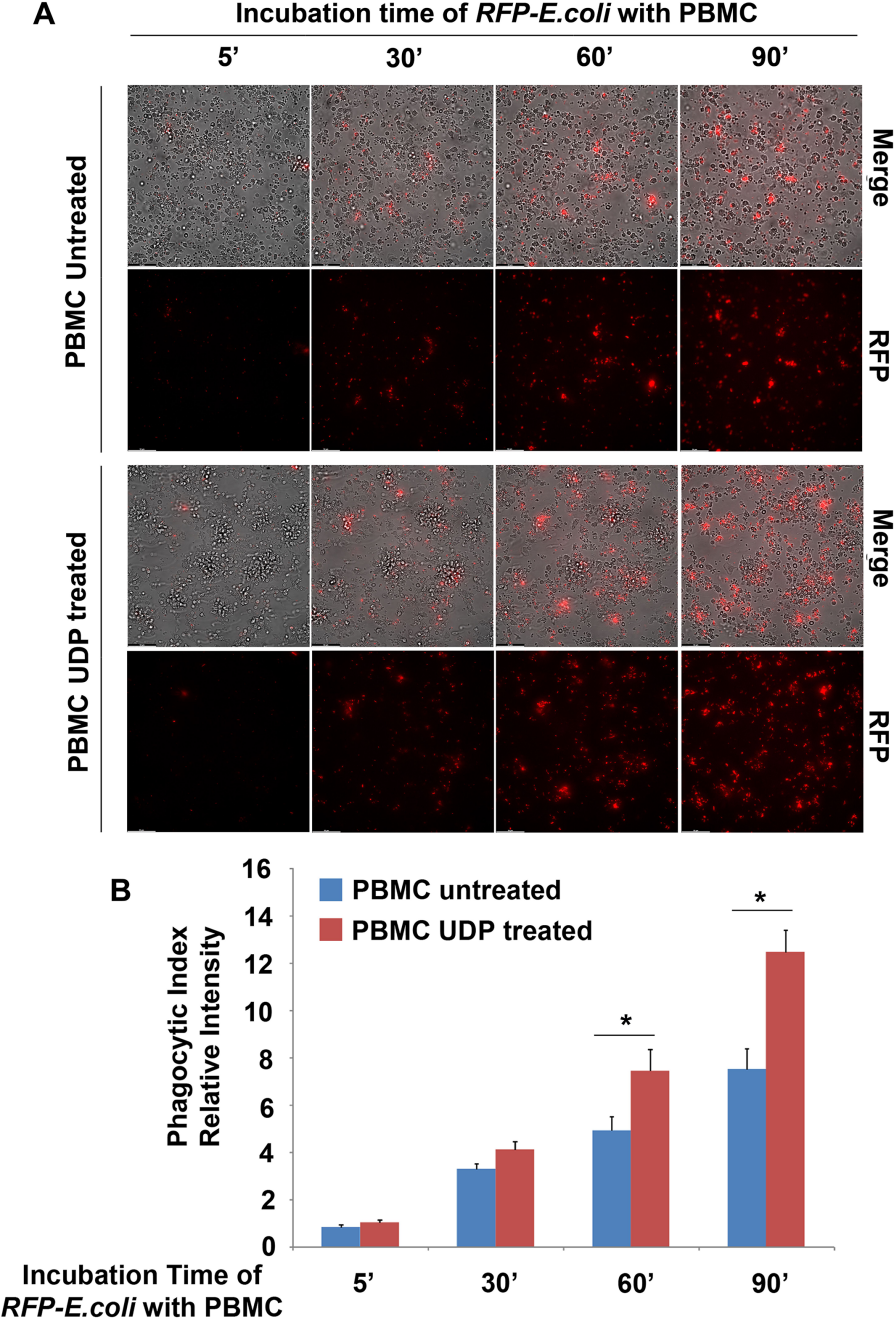
UDP enhances bacterial phagocytosis by PBMCs. **(A)** DH5α *E. coli* bacterial cells, stably transformed with the pCMV6-AC-RFP Mammalian Expression Vector (Origene), were grown in LB broth at 37°C until reaching mid-logarithmic phase (OD_600_ = 0.6). Fifty microliters of the bacterial suspension (OD_600_ = 0.6) were incubated with 5 × 10^6^ PBMCs, pretreated or not with 100 µM UDP for 2 h. Representative time-lapse videos of PBMC-bacteria co-cultures in the presence or absence of UDP are provided as Videos 1 (Not treated) and 2 (UDP-treated). Magnification 20X. **(B)** Phagocytosis was assessed at the indicated time points (5, 30, 60, and 90 min) by calculating the Phagocytic Index, defined as the ratio between intracellular red fluorescence and the total number of cells. Intracellular red fluorescence, corresponding to bacterial uptake, was quantified using ImageJ. Data are expressed as mean ± SE from three independent experiments. Statistical analysis was performed using Student’s t-test, p < 0.01. The asterisk indicates a statistically significant difference according to the Student's t-test (p < 0.05).

**Figure 5:**
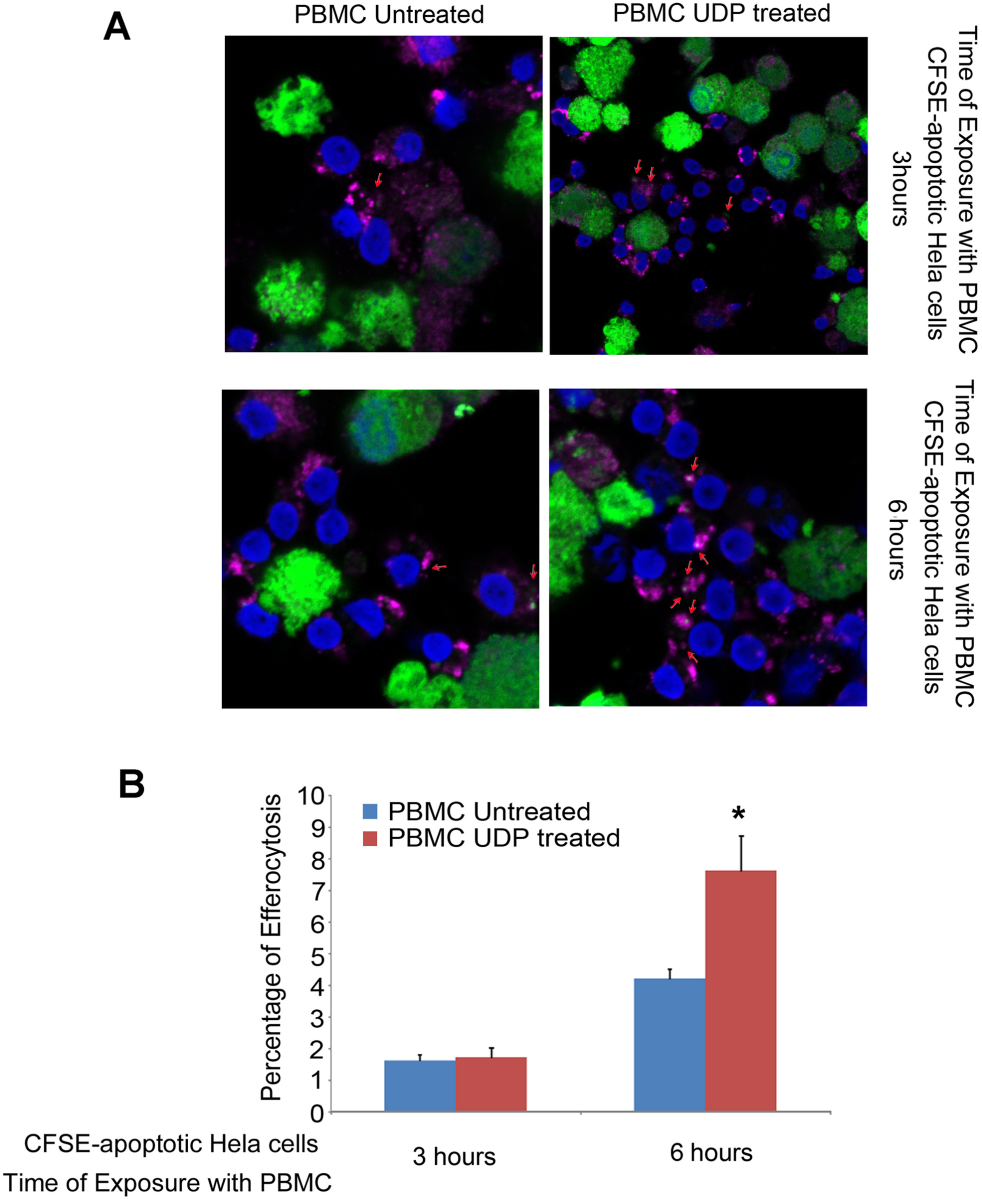
UDP enhances efferocytosis of apoptotic HeLa cells by PBMCs. **(A)** Apoptotic HeLa cells, generated by 3-days starvation in HBSS and labeled with CFSE (1 µM), were incubated with PBMCs (5 × 10^6^), pretreated or not with 100 µM UDP for 2 h. Co-cultures were maintained in the presence or absence of UDP for 3 or 6 h. Efferocytosis was visualized by immunofluorescence microscopy through co-localization of CFSE-positive apoptotic bodies (green) within LAMP-1^+^ lysosomal compartments (magenta). Nuclei were counterstained with DAPI (blue). Magnification 63X. **(B)** Quantification of efferocytosis was expressed as the percentage of PBMCs displaying LAMP-1^+^ vesicles containing CFSE^+^ apoptotic material. Data represent mean ± SE of three independent experiments. Statistical significance was assessed by Student’s t-test, p < 0.01. The asterisk indicates a statistically significant difference according to the Student's t-test (p < 0.05).

Next, we investigated whether extracellular UDP could also modulate the phagocytic function of peripheral blood mononuclear cells (PBMCs). Phagocytosis represents a critical effector function of myeloid cells, encompassing both the internalization of pathogens and the clearance of apoptotic cells, a process referred to as efferocytosis. Since these activities are closely linked to the acquisition of a differentiated myeloid phenotype, we hypothesized that UDP might enhance both forms of phagocytosis. To evaluate bacterial phagocytosis, PBMCs were incubated with Escherichia coli DH5α expressing red fluorescent protein (RFP), in the presence or absence of 100 µM UDP. Prior to bacterial addition, cells were pretreated with UDP for 2 hours. The PBMCs were then incubated with the bacterial suspension (OD_600_ = 0.6) and collected at multiple time points (5, 30, 60, and 90 minutes) by fluorescence microscopy analysis of RFP fluorescence, which indicates bacterial uptake. While UDP treatment did not significantly affect phagocytosis at early time points, a marked increase in bacterial internalization was observed at 60 and 90 minutes. In particular, after 90 minutes of co-incubation, UDP-treated PBMCs exhibited nearly a two-fold increase in phagocytic capacity compared to untreated controls, as shown by the elevated RFP signal intensity (**Figures 4A, B**, **Supplementary Videos S1** (Not treated) and 2 (UDP-treated)). We next assessed the ability of PBMCs to perform efferocytosis. Apoptotic HeLa cells were labeled with CFSE and co-cultured with PBMCs for either 3 or 6 hours, in the presence or absence of UDP. After incubation, cells were fixed and stained for LAMP1, a lysosomal membrane protein used to mark phago-lysosomal compartments. Confocal microscopy was used to analyze the colocalization between CFSE and LAMP1 signals, indicative of successful efferocytic events. While little colocalization was observed after 3 hours, a substantial increase in CFSE^+^LAMP1^+^ vesicles was detected after 6 hours of co-culture, specifically in the presence of UDP. Quantification of these events revealed a statistically significant enhancement of efferocytosis—approximately two-fold—upon UDP treatment compared to control conditions (**Figures 5A, B**). These findings demonstrate that UDP not only promotes monocyte differentiation toward a dendritic phenotype but also functionally enhances key innate immune effector responses such as bacterial phagocytosis and efferocytosis. These results support the notion that UDP acts as an immunomodulatory metabolite capable of shaping myeloid cell behavior and clearance functions, potentially contributing to tissue homeostasis and resolution of inflammation.

### Transcriptomic profiling reveals UDP-driven immunomodulatory gene signatures in PBMCs

**Figure 6:**
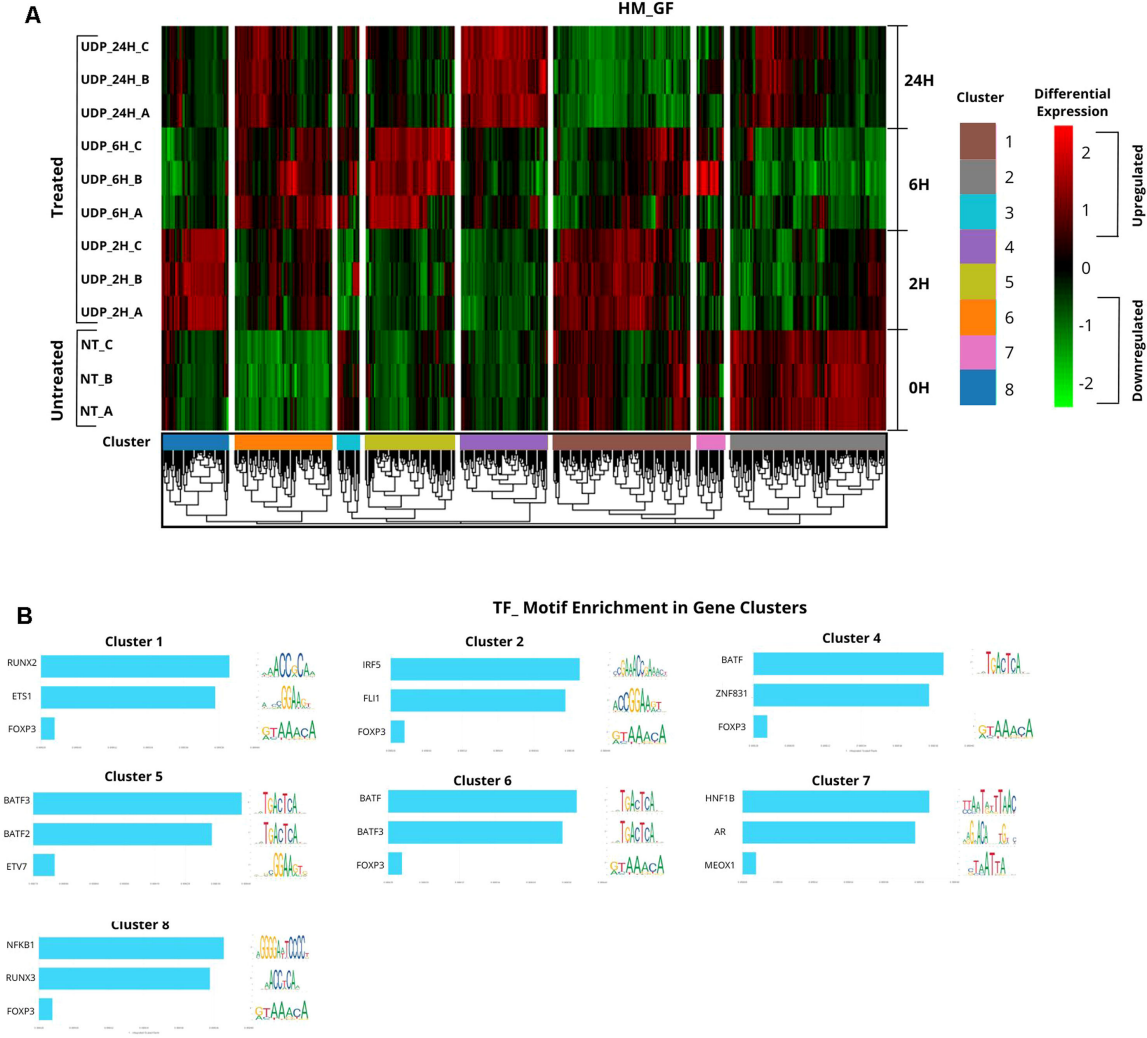
Transcriptomic profiling of PBMCs stimulated with UDP. Freshly isolated PBMCs (1 × 10^6^) were stimulated with 100 µM UDP for 2 h, 6 h, or 24 h, alongside unstimulated controls, in triplicate. Total RNA was extracted and analyzed using the Nanostring nCounter^®^ Immunology Panel, which interrogates the expression of more than 500 immune-related genes. **(A)** Heatmap of differentially expressed genes across time points shows distinct transcriptional signatures induced by UDP stimulation. Data represent normalized counts (z-score). Relative expression levels are shown in red (upregulation) and green (downregulation). Genes were clustered by hierarchical clustering (Euclidean distance), while samples are annotated by experimental condition. **(B)** Transcriptional motifs enrichment in the different gene clusters was analyzed by ChIP-X Enrichment Analysis 3. Bar charts represent the top 3 scaled TF ranks from integrated rank score for each cluster. For each TF sequence logo representation of the binding specificity are reported (https://jaspar.elixir.no/).

To gain deeper insight into the molecular effects of extracellular UDP on immune cells, we performed a transcriptomic analysis of human peripheral blood mononuclear cells (PBMCs) exposed to UDP. Previous results had shown that UDP profoundly affects monocyte proliferation and promotes their differentiation into dendritic-like cells, suggesting that it may act as a modulator of innate immune cell fate and function. However, the broader transcriptional programs engaged by UDP stimulation in PBMCs remained undefined. To address this, we stimulated freshly isolated PBMCs with 100 µM UDP for different durations, 2 hours, 6 hours, and 24 hours—and compared them to unstimulated controls. Total RNA was extracted from triplicate samples for each condition, and gene expression profiling was carried out using the NanoString nCounter Human Immunology Panel, which quantifies the expression of over 500 genes associated with immune activation, regulation, cell differentiation, cytokine signaling, antigen presentation, and inflammation. This time-course approach was designed to capture both early and late transcriptional responses to UDP and to help distinguish immediate signaling events from downstream effects linked to cellular differentiation or functional reprogramming. By integrating this transcriptomic dataset with our previous phenotypic observations, we aimed to identify specific gene networks and pathways potentially responsible for the UDP-induced inhibition of monocyte proliferation, the acquisition of dendritic-like markers, and the enhanced phagocytic activity observed in our earlier experiments. To further dissect the transcriptional programs induced by UDP, we performed unsupervised clustering of all differentially expressed genes (DEGs) detected at 2 h, 6 h, and 24 h following stimulation (**Supplementary Table S1**). This analysis revealed eight distinct gene clusters characterized by unique temporal expression patterns (**Figure 6A**, **Supplementary Table S2**). Cluster 1 consisted of genes transiently upregulated at early time points (2 h and 6 h), returning to basal levels at 24 h. This cluster included several antigen presentation and adaptive immune response genes such as HLA-DPA1, HLA-DPB1, CD4, CD74, as well as inflammatory mediators like IL1B, IL6, CXCL1, CXCR4, and regulators of T-cell signaling (ZAP70, STAT4). The early induction of these genes suggests a rapid activation of innate-to-adaptive immune crosstalk pathways upon UDP stimulation. Cluster 2 was strongly downregulated at early time points and included genes critical for monocyte and macrophage activation (CD14, CCR1, CSF1R), components of the complement pathway (C1QA, C3, C2), and various receptors involved in myeloid cell recognition (TLR4, CD36, CD40LG). This indicates a rapid suppression of classical monocyte programs, consistent with our flow cytometry results showing reduced monocyte proliferation. Cluster 3 included relatively few genes with low expression and limited temporal variation (e.g., AHR, ITGAE, NCR1), suggesting a minor contribution to UDP-induced immune reprogramming. Cluster 4 showed late upregulation (24 h) and contained multiple genes linked to T-cell activation and exhaustion, including TIGIT, HAVCR2 (TIM-3), LAG3, CTLA4, as well as effector molecules like Granzyme B (GZMB) and IL17F. The delayed induction of this cluster suggests that UDP not only primes early innate immune pathways but also influences adaptive immune responses, possibly driving activation-to-exhaustion transitions. Cluster 5 genes were transiently upregulated at 6 h, including cytokines (IL2, IL21, IL26), transcriptional regulators (IRF4, IRF7), and markers of T-helper cell polarization (FOXP3, ICOS). This indicates an intermediate wave of transcriptional reprogramming that may bridge early innate activation and later adaptive immune remodeling. Cluster 6 contained genes persistently upregulated from early to late time points, such as IFNG, IL10, CCL5, CCR7, CD80, CD274 (PD-L1), and interferon-related genes (IDO1, STAT6). This stable induction highlights a sustained activation state characterized by both pro- and anti-inflammatory mediators, suggesting a balanced immunoregulatory output. Clusters 7 and 8 were less populated but showed interesting dynamics. Cluster 7 genes (e.g., CX3CL1, IL32, TLR7) exhibited modest regulation with specialized functional roles in chemotaxis and antiviral signaling. Cluster 8 genes were sharply upregulated at 2 h and quickly returned to baseline, including TNF, CCL3, CCL4, CXCL10, ICAM1, representing a classic early inflammatory response. Overall, these findings indicate that UDP triggers a highly dynamic, multi-phase transcriptional program in PBMCs. Early changes (Clusters 1, 8) reflect rapid innate immune activation, including chemokine/cytokine signaling and antigen presentation. Mid-phase responses (Clusters 5, 6) consolidate adaptive and regulatory signatures, while late changes (Cluster 4) suggest the engagement of T-cell exhaustion and long-term immune adaptation mechanisms. To gain mechanistic insights into the regulatory networks underlying the UDP-induced transcriptional response, we performed a transcription factor target over-representation analysis of genes belonging to each expression cluster (**Figure 6B**). This analysis revealed distinct enrichment patterns of transcription factor (TF) binding motifs, suggesting that UDP stimulation engages different upstream regulatory modules depending on the temporal gene expression pattern. Promoters of Cluster 2 genes, which were strongly downregulated at early time points, were significantly enriched for IRF5 and FLI1 motifs. IRF5 is a key regulator of innate immune responses and macrophage activation, whereas FLI1 is implicated in hematopoietic lineage development and endothelial signaling. The reduced expression of Cluster 2 genes following UDP stimulation may reflect an inhibition of IRF5/FLI1-driven transcriptional programs associated with classical monocyte activation and myeloid lineage commitment. In contrast, Clusters 4, 5, and 6, which contained genes upregulated at intermediate to late time points (including T cell activation and exhaustion markers such as TIGIT, HAVCR2, LAG3, CTLA4, IFNG, FOXP3, CD274), showed significant enrichment for binding sites of the BATF family of transcription factors. BATF proteins are well-known regulators of effector and exhausted T cell differentiation, suggesting that UDP stimulation induces a BATF-driven program favoring adaptive immune remodelling and long-term functional adaptation. Promoters of Cluster 1 genes, which were transiently upregulated at 2–6 h, were enriched for RUNX2 and ETF binding motifs. RUNX2 is classically associated with osteogenic differentiation but also plays roles in dendritic cell maturation and immune regulation, whereas ETF (electron-transfer flavoprotein)–associated motifs point to potential links between metabolic reprogramming and transcriptional control. Cluster 3, consisting of a small number of genes with minimal differential expression, did not show any statistically significant enrichment for TF motifs, likely due to its limited gene representation. Promoters of Cluster 7 genes, which were modestly regulated, were enriched for HNF1B and AR motifs, transcription factors more typically linked to epithelial biology and hormonal regulation. This may reflect cell-type–specific responses in rare PBMC subsets or tissue-resident cell signatures. Finally, Cluster 8, which included early and transiently upregulated genes such as TNF, CCL3, CCL4, CXCL10, ICAM1, showed a clear enrichment for NF-κB and RUNX3 motifs. NF-κB is a canonical driver of early inflammatory gene expression, consistent with the rapid induction of pro-inflammatory cytokines and chemokines following UDP exposure, while RUNX3 is a known regulator of cytotoxic T cell identity and NK cell development. These data indicate that UDP stimulation not only triggers distinct temporal transcriptional modules but also selectively engages specific upstream transcription factor families, including NF-κB, RUNX3, BATF, and IRF5, depending on the functional context of each gene cluster. This suggests that UDP acts as a multifaceted immune modulator capable of orchestrating a complex regulatory network involving both innate and adaptive immune transcriptional circuits.

## Discussion

Our findings reveal that extracellular UDP profoundly shapes the biology of human peripheral blood mononuclear cells (PBMCs), acting as a key immunomodulatory metabolite that coordinates proliferation, differentiation, and effector functions of myeloid and lymphoid subsets. We demonstrate that UDP suppresses the proliferative activity of CD14^+^ monocytes (**Figures 1A, B**) and promotes their differentiation into dendritic-like cells within PBMC (**Figures 2A, B**). To determine whether this effect was directly mediated by UDP on monocytes rather than indirectly through other immune populations within PBMCs, we next analyzed the differentiation process using purified CD14^+^ monocytes. When cultured under standard conditions promoting monocyte-to-dendritic cell differentiation (IL-4 and GM-CSF), UDP treatment resulted in a marked loss of CD14 expression and a strong increase in the population of CD14^-^CD16^-^HLA-DR^+^CD11c^+^ cells (**Figures 3A-C**). These findings confirm that UDP directly enhances the differentiation of monocytes into immature dendritic cells (moDCs), independently of paracrine or accessory cell effects within mixed PBMC cultures. This observation aligns with previous reports indicating that purinergic signaling can modulate myeloid cell differentiation and activation states (24). In particular, P2RY6 activation has been linked to the regulation of cytokine production, NF-κB signaling, and lysosomal biogenesis in macrophages and microglia (18, 19, 25). Our data suggest that UDP may similarly reprogram human monocytes toward a more antigen-presenting, pro-phagocytic phenotype. The observed downregulation of CD14, together with increased HLA-DR and CD11c expression, reflects a transition from a monocyte identity toward a dendritic lineage, a process typically associated with enhanced antigen processing and T-cell stimulatory capacity (26). Mechanistically, this effect could involve UDP-dependent activation of intracellular pathways such as ERK and NF-κB, which are known to drive dendritic cell maturation and functional polarization (4). Collectively, these results support a model in which extracellular UDP acts as a differentiation cue for circulating monocytes, potentially linking metabolic stress or tissue damage to the expansion of antigen-presenting cell populations in peripheral blood and inflamed tissues. Because our data suggest that UDP may reprogram human monocytes toward a more antigen-presenting, pro-phagocytic phenotype, we next investigated whether UDP stimulation could also enhance functional aspects of phagocytosis and efferocytosis. To this end, we evaluated both bacterial uptake and the clearance of apoptotic cells. We found that UDP-treated PBMCs displayed a markedly increased capacity to internalize E. coli DH5α bacteria expressing RFP, with a nearly two-fold rise in the phagocytic index compared to untreated controls (**Figures 4A, B**). Similarly, UDP significantly enhanced the efferocytic activity of PBMCs, as demonstrated by the increased colocalization of apoptotic CFSE-labeled HeLa cells with the lysosomal marker LAMP1 after 6 hours of co-culture (**Figures 5A, B**). These findings indicate that extracellular UDP not only promotes the differentiation of monocytes into dendritic-like cells but also potentiates key effector functions involved in immune clearance and tissue homeostasis. This is consistent with previous evidence showing that P2RY6 signaling enhances phagocytic and lysosomal activity in microglia and macrophages (27), and that UDP release acts as a “find-me” or “eat-me” signal during tissue injury and neuroinflammation (28–30). The observed enhancement of bacterial and apoptotic cell engulfment suggests that UDP acts as a metabolic cue linking purinergic signaling to innate immune effector activation. Altogether, these results reinforce the notion that UDP functions as an immunoregulatory metabolite capable of integrating environmental stress and immune activation to modulate myeloid cell behavior. Transcriptomic profiling further uncovered complex, time-dependent gene expression programs governed by distinct transcriptional modules, highlighting UDP’s pleiotropic role in regulating immune cell fate and function (**Figures 6A, B**, **Supplementary Table S1**). The observed inhibition of CD14^+^ monocyte proliferation upon UDP treatment suggests that UDP–P2RY6 signaling may function as a checkpoint that enforces a quiescent or differentiative state within the myeloid compartment. This aligns with previous studies showing that P2Y6 activation can modulate macrophage polarization and microglial activation, promoting a shift from proliferative to functional phenotypes (17, 31). The concomitant increase in HLA-DR^+^CD11c^+^ dendritic-like cells supports the notion that UDP promotes monocyte-to-dendritic differentiation rather than merely suppressing cell division. In cytokine-driven differentiation assays (IL-4/GM-CSF), UDP further accelerated the transition from CD14^+^ to CD14^-^CD11c^+^HLA-DR^+^ cells, confirming a direct role in promoting the acquisition of antigen-presenting cell characteristics (**Figure 3**). Consistently, UDP-treated monocyte-derived cells also displayed increased expression of dendritic cell maturation markers, including CD80 and CD40 (**Figures 3D, E**), supporting the acquisition of a more functionally competent dendritic cell-like state. These results suggest that UDP acts as a molecular cue that guides monocyte fate decisions, potentially linking extracellular nucleotide signaling to immune homeostasis and tissue remodeling. Previous studies have demonstrated that soluble immunomodulatory factors can profoundly influence the maturation and functional properties of monocyte-derived dendritic cells in a context- and dose-dependent manner. For example, Galectin-1 has been shown to differentially regulate MoDC phenotype and cytokine production, modulating the expression of co-stimulatory molecules such as CD80 and CD86 and shaping cytokine profiles associated with either immune activation or immune suppression, depending on its concentration (32). In this framework, our data suggest that UDP represents an additional extracellular signal capable of modulating monocyte-derived dendritic cell differentiation, supporting the concept that non-cytokine soluble mediators contribute to fine-tuning dendritic cell maturation and immune function. Functionally, UDP enhanced both bacterial phagocytosis and efferocytosis, indicating a broader role in regulating myeloid effector mechanisms. The increased uptake of RFP-expressing E. coli and apoptotic HeLa cells by UDP-treated PBMCs supports the idea that UDP not only induces differentiation toward phagocytic phenotypes but also primes these cells for enhanced clearance functions. These findings are consistent with previous reports showing that UDP–P2Y6 signaling can promote phagocytosis through calcium-dependent cytoskeletal rearrangements and lysosomal activation (18, 30). The ability of UDP to simultaneously limit proliferation and enhance phagocytic function reflects a coordinated reprogramming of myeloid cell behavior, reminiscent of the transition from inflammatory monocytes to tissue-resident macrophages or dendritic cells engaged in resolution of inflammation. Transcriptomic profiling of PBMCs treated with UDP revealed a highly dynamic and temporally ordered response involving early innate activation, intermediate adaptive remodeling, and late regulatory adaptation (**Figures 6A, B**, **Supplementary Tables S1**, **S2**). Early gene clusters (1 and 8) were dominated by NF-κB–driven inflammatory genes, including TNF, CCL3, CCL4, and CXCL10, indicating rapid engagement of canonical danger-signaling pathways (33, 34). In contrast, Cluster 2, which was downregulated at early time points, contained genes associated with classical monocyte activation (CD14, CSF1R, TLR4), suggesting that UDP rapidly suppresses macrophage-like transcriptional programs, consistent with the phenotypic inhibition of monocyte proliferation. The sustained upregulation of Clusters 5 and 6, enriched for IFNG, IL10, CD80, and PD-L1, reflects a balanced activation state combining pro- and anti-inflammatory mediators (32, 35), while the late induction of Cluster 4 genes (TIGIT, LAG3, CTLA4, GZMB, IL17F) indicates engagement of T-cell activation and exhaustion pathways (36). Together, these findings depict UDP as a multifaceted regulator capable of orchestrating both innate and adaptive immune programs across distinct temporal phases. Promoter enrichment analysis provided further insight into the transcriptional architecture underlying these responses. Early upregulated genes were enriched for NF-κB and RUNX3 motifs, confirming the involvement of canonical inflammatory and cytotoxic T/NK cell transcriptional drivers. Mid-to-late clusters (4–6) showed enrichment for BATF family motifs, consistent with transcriptional programs associated with T-cell activation and exhaustion. Conversely, Cluster 2, which was repressed early, displayed enrichment for IRF5 and FLI1 motifs, suggesting inhibition of classical monocyte and macrophage activation pathways (37). The transcriptomic analysis performed in this study was intended to provide an exploratory overview of the immune-related transcriptional changes induced by UDP treatment. Accordingly, these data should be interpreted as hypothesis-generating rather than as a definitive mechanistic dissection. The observed transcriptional signatures are consistent with the phenotypic and differentiation data and support a role for UDP in modulating monocyte fate toward a dendritic cell like phenotype. Further studies employing time-resolved and cell subtype specific approaches will be required to fully elucidate the underlying molecular mechanisms. Collectively, these results highlight that UDP modulates distinct transcriptional circuits depending on the phase of stimulation, initially activating inflammatory gene expression, then promoting differentiation and immune regulation. Altogether, our study identifies UDP as a potent immunomodulatory signal capable of integrating metabolic and transcriptional cues to control myeloid differentiation, phagocytic capacity, and global immune reprogramming. By driving a shift from proliferative to functionally specialized states, UDP may contribute to the resolution of inflammation and maintenance of immune homeostasis. Given the emerging roles of extracellular nucleotides in cancer, neuroinflammation, and infection, the UDP–P2RY6 axis represents a promising target for therapeutic modulation of innate and adaptive immune responses.

## Data Availability

The datasets presented in this study can be found in online repositories [Gene Expression Omnibus (GEO)]. The names of the repository/repositories and accession number(s) can be found below: GSE315661 (GEO).
