# Correction: Uridine diphosphate drives myeloid differentiation and functional reprogramming through dynamic transcriptional network

**DOI:** 10.3389/fimmu.2026.1811262

**Published:** 2026-02-20

**Authors:** Caterina Giordano, Debora Gentile, Emilio Straface, Raffaella Gallo, Costanza Maria Cristiani, Antonio Abatino, Arianna Pastore, Marilena Celano, Alessandro Arcucci, Francesco Albano, Geppino Falco, Claudia Veneziano, Gianluca Santamaria, Ilenia Aversa, Lisa Isdraele Romano, Camillo Palmieri, Giuseppe Fiume

**Affiliations:** 1 Department of Experimental and Clinical Medicine, University of Catanzaro “Magna Graecia”, Catanzaro, Italy; 2 Neuroscience Research Center, Department of Medical and Surgical Sciences, University Magna Graecia of Catanzaro, Catanzaro, Italy; 3 Department of Pharmacy, University of Naples Federico II, Naples, Italy; 4 Department of Life Sciences, University of Catanzaro “Magna Graecia”, Catanzaro, Italy; 5 Department of Public Health, University of Naples “Federico II”, Naples, Italy; 6 Department of Biology, University of Naples “Federico II”, Naples, Italy

**Keywords:** dendritic cells, innate immunity, monocyte differentiation, purinergic signaling, uridine diphosphate (UDP)

Affiliation “Neuroscience Research Center, Department of Medical and Surgical Sciences, University Magna Graecia of Catanzaro, Catanzaro, Italy” was omitted for author “Costanza Maria Cristiani”. This affiliation has now been added for author “Costanza Maria Cristiani”.

The original version of this article has been updated.

