# Supplementary material for: Uridine diphosphate drives myeloid differentiation and functional reprogramming through dynamic transcriptional network
Source: Front Immunol. 2026 Jan 30;17:1743389. doi: 10.3389/fimmu.2026.1743389 (PMC12901406; doi:10.3389/fimmu.2026.1743389)
Supplement: Supplementary file 3 [file DataSheet1.docx]

**
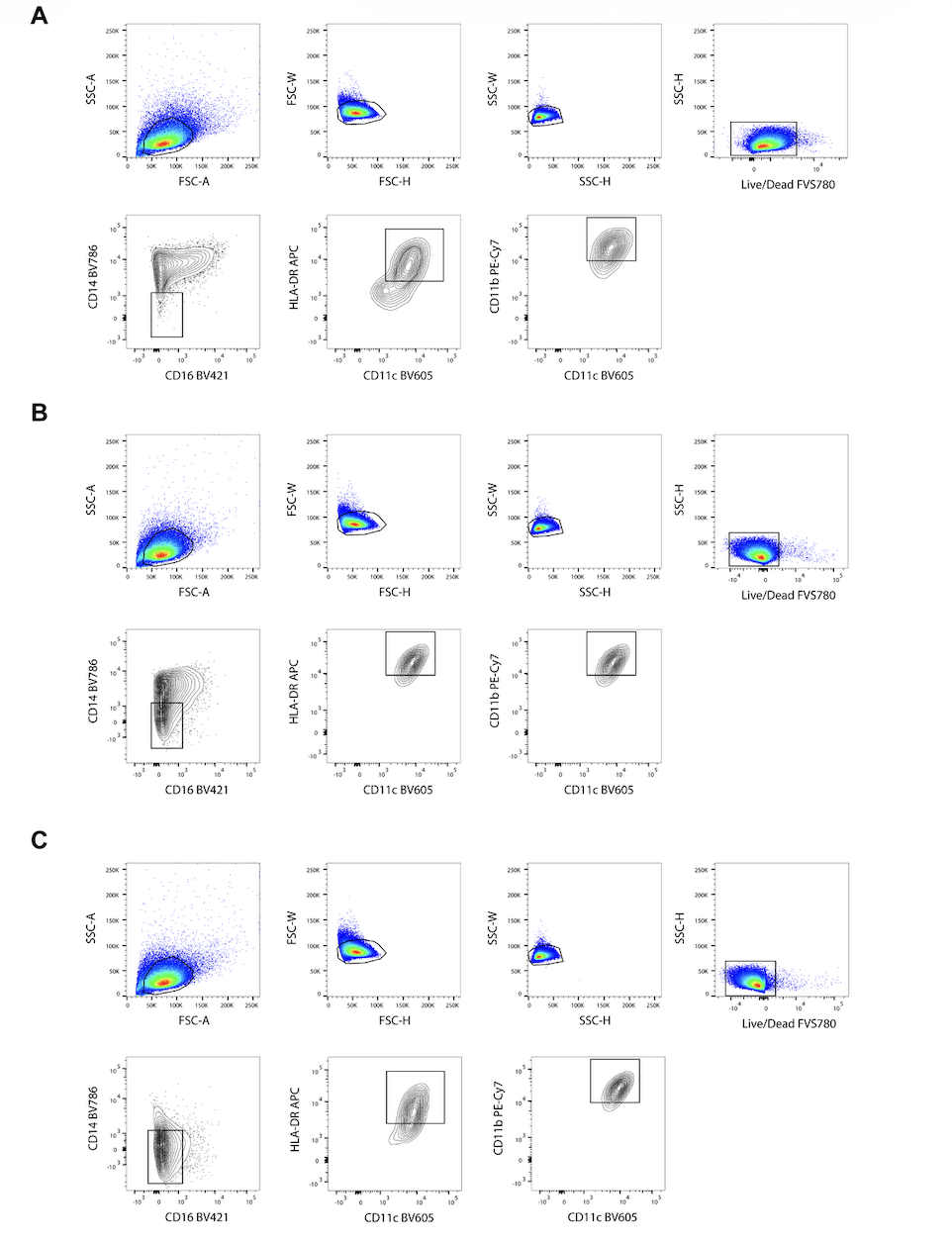
**

**Supplementary Figure 1. Representative flow cytometry gating strategy used to identify monocyte-derived dendritic cells (moDCs) and assess activation marker expression under different differentiation conditions.** (A) Control condition: monocytes cultured with GM-CSF alone. (B) Monocytes differentiated with GM-CSF and IL-4. (C) Monocytes differentiated with GM-CSF, IL-4, and UDP. Sequential gating steps included selection of the cell population based on forward and side scatter (FSC/SSC) parameters, doublet exclusion (singlet discrimination), and removal of dead cells. moDCs were subsequently identified as CD14⁻CD16⁻HLA-DR⁺CD11c⁺ cells.
